# Oral administration of a new copper (I) complex with coumarin as ligand: modulation of the immune response and the composition of the intestinal microbiota in *Onchorhynchus mykiss*

**DOI:** 10.3389/fchem.2024.1338614

**Published:** 2024-05-14

**Authors:** Mick Parra, Maialen Aldabaldetrecu, Pablo Arce, Sarita Soto-Aguilera, Rodrigo Vargas, Juan Guerrero, Mario Tello, Brenda Modak

**Affiliations:** ^1^ Laboratory of Natural Products Chemistry, Centre of Aquatic Biotechnology, Faculty of Chemistry and Biology, University of Santiago of Chile, Santiago, Chile; ^2^ Laboratory of Bacterial Metagenomic, Centre of Aquatic Biotechnology, Faculty of Chemistry and Biology, University of Santiago of Chile, Santiago, Chile; ^3^ Laboratory of Coordination Compounds and Supramolecularity, Faculty of Chemistry and Biology, University of Santiago of Chile, Santiago, Chile; ^4^ Aquaculture Production Unit, Universidad de Los Lagos, Osorno, Chile

**Keywords:** coumarin, copper (I) complex, intestinal microbiota, salmonids, onchorhynchus mykiss, immune system

## Abstract

[Cu(NN_1_)_2_]ClO_4_ is a copper (I) complex, where NN_1_ is an imine ligand 6-((quinolin-2-ylmethylene) amino)-2H-chromen-2-one obtained by derivatization of natural compound coumarin, developed for the treatment of infectious diseases that affect salmonids. In previous research, we showed that the Cu(I) coordination complex possesses antibacterial activity against *Flavobacterium psychrophilum*, providing protection against this pathogen in rainbow trout during challenge assays (with an RPS of 50%). In the present study, the effects of administering [Cu(NN_1_)_2_]ClO_4_ to *Oncorhynchus mykiss* over a 60-days period were evaluated with regard to systemic immune response and its potential to alter intestinal microbiota composition. In *O. mykiss*, an immunostimulatory effect was evident at days 30 and 45 after administration, resulting in an increment of transcript levels of IFN-γ, IL-12, TNF-α, lysozyme and perforin. To determine whether these immunomodulatory effects correlated with changes in the intestinal microbiota, we analyzed the metagenome diversity by V4 16S rRNA sequencing. In *O. mykiss*, both [Cu(NN_1_)_2_]ClO_4_ and commercial antibiotic florfenicol had comparable effects at the phylum level, resulting in a predominance of proteobacteria and firmicutes. Nonetheless, at the genus level, florfenicol and [Cu(NN_1_)_2_]ClO_4_ complex exhibited distinct effects on the intestinal microbiota of *O. mykiss*. In conclusion, our findings demonstrate that [Cu(NN_1_)_2_]ClO_4_ is capable of stimulating the immune system at a systemic level, while inducing alterations in the composition of the intestinal microbiota in *O. mykiss*.

## 1 Introduction

In Chile, the aquaculture industry occupies an important place within the country’s economy, where salmon farming is the main sector, being the second largest producer worldwide after Norway (FAO, 2022). Favorable climatic and environmental conditions have generated rapid growth in this productive sector, with Atlantic salmon, coho salmon and rainbow trout being the main species produced (Sernapesca, 2023a). However, this growth in production has generated an increase in outbreaks of infectious diseases, because fish are exposed to constant stress due to handling, transportation, higher density, changes in temperature and salinity among others, where these conditions increase their susceptibility to different types of pathogens, which cause significant economic losses to the industry (Mohapatra et al., 2013). The pathogens that most affect the salmon industry are bacteria, mainly *Piscirickettsia salmonis*, *Tenacibaculum dicentrarchi* and *Flavobacterium psycrhophilum* (Sernapesca, 2023a). In the case of *P. salmonis*, this bacterium has been the infectious agent that has caused the most deaths in salmonids for more than 30 years; it is estimated that the losses associated with this pathogen are in range between US$700 to US$800 million annually (Maisey et al., 2017).

To control diseases caused by these bacteria, vaccines and antibiotics are mainly used. However, due still to little knowledge of the immune system in teleosts, the vaccines have not been effective (Maisey et al., 2017; Figueroa et al., 2022). Because vaccines are not sufficient to control the diseases caused by these bacteria, the Chilean salmon industry uses a large amount of antibiotics. By 2022, 341.5 tons will be used, of which about 97.7% will be used in seawater and 2.3% in freshwater. On the other hand, 97.84% of the antibiotics used in seawater correspond to florfenicol, while in freshwater 73.6% of the antibiotic used was oxytetracycline (Sernapesca, 2023b). In seawater, 91.28% of the antibiotics were used against *P. salmonis* and 1.52% against *T. dicentrarchi*, while in fresh water 39.62% of the antibiotic used was against *Flavobacterium psychrophilum*. This large amount of antibiotic used has caused various problems, such as microbial resistance (Henríquez et al., 2016; Saavedra et al., 2017). On the other hand, environmental pollution is generated, because antibiotics are administered to the salmon through food, so it leaks from the cages into the water and marine sediment in which they are found. In addition, there is a flow of traces of antibiotics present in salmon urine and feces into the marine environment (Quiñones et al., 2019; Hossain et al., 2022). Another problem of excessive use of antibiotics, reported in recent years, is the effect on the composition of the intestinal microbiota of salmonids. Antibiotics generate dysbiosis in the intestine of fish, which causes changes in various biological processes, including the immune response (Donati et al., 2022; Huyben et al., 2023; Xavier et al., 2023). The problem of excessive use of antibiotics has been recognized by Chilean salmon producers, so in 2019, the salmon farming sector and the Monterey Bay Aquarium launched the Chilean Salmon Antibiotic Reduction Program (CSARP) with the aim of reducing the use of antibiotics by 50% by 2025. Considering the need to look for alternatives to the use of antibiotics in the salmon industry, various products with antibacterial and immunostimulant capacity have been studied, with the aim of reducing the use of antibiotics without increasing the mortalities generated by the pathogens that affect the industry. One of the alternatives is the use of compounds of natural origin, which when administered in diets, cause positive effects in the fish organism. For example, a study with 7-diethylamino-4-methylcoumarin in zebrafish showed that dietary coumarin administration increased growth of the fish (Blanc et al., 2020). Other studies have shown that the flavonol quercetin improves growth and immune response in grass carp (*C. idella*) (Xu et al., 2019), while in fish zebra (*D. rerio*) improves immunity, increases antioxidant capacity and the resistance against *Aeromonas hydrophila* (Wang et al., 2020). Rutin has also been administered in fish, and it has been observed that in rainbow trout (*Onchorhynchus mykiss*) it reduces liver damage induced by oxytetracycline and oxidative stress (Nazeri et al., 2017). However, the administration of oral treatments not only generates effect at a systemic level in the fish, also directly affects the intestine, causing a change in the composition of the intestinal microbiota. This phenomenon is of radical importance, because the microbiota plays a fundamental role in the health of the host, modulating the immune response and metabolism, in addition to generating protection against different infectious agents, while an imbalance in its composition could considerably affect the host (Brugman et al., 2018). The importance of maintaining a balance in the composition of the microbiota is because the beneficial microorganisms that colonize the mucous membranes of animals constitute a well-conserved interaction that contributes to the modulation of the immune system, which implies a tolerance to these beneficial microorganisms while pathogenic microorganisms are combated (Gomez et al., 2013). In fish, the mucous membranes are colonized by a wide variety of microorganisms (Gomez et al., 2013; Salinas, 2015), which interact with the immune system by modulating various components, such as the number of neutrophils present in the intestine (Bates et al., 2007), promoting cell proliferation (Cheesman et al., 2011), increasing IgT production by B lymphocytes and regulating cytokine production (López Nadal et al., 2020). In this context, it has been studied that the administration of compounds of natural origin as a dietary supplement also generates an effect on the composition of the intestinal microbiota of fish. In this perspective, it has been shown that resveratrol in tilapia (*Oreochromis niloticus*) positively modulates the composition of the microbiota, significantly enriching the phylum firmicutes, and the proportion of beneficial microbial taxa such as Acetobacteraceae and Methylobacteriaceae, while decreasing the proportion of harmful microbial taxa such as Streptococcaceae, Rickettsiaceae, Clostridiaaceae, Parachlamydiaceae, Mycobactereaceae, Lachnospiraceae and Rhodospirillales (Zheng et al., 2018). In the case of daidzein administration in juvenile turbot (*S. maximus*) an increase in the relative abundance of beneficial bacteria such as *Lactobacillus* and *Bifidobacterium* was observed, while potential pathogenic bacteria such as *Pseudomonas, Acinetobacter* and *Helicobacter* decreased (Ou et al., 2019). However, although natural compounds have multiple positive effects, currently, there is a great interest in copper coordination compounds based on ligands with N- donor atom. This interest arises from its properties antiproliferative, antibacterial, antiviral, and cytotoxic (Krasnovskaya et al., 2020), as well as its ability to modulate the immune response (Djoko et al., 2018; Liu et al., 2022). The adaptability of transition metal compounds, in particular Cu(I) coordination complexes with bidentate nitrogen donor ligands, is well known. Therefore, several researches have been conducted towards the controlled obtaining of its electrochemical properties through the selection of its ligands (Cáceres-Vásquez et al., 2023). For all the aforementioned problems and considering the need to generate alternatives to antibiotics, in addition to the positive effect that natural compounds and their derivatives have, added to the properties that transition metal complexes possess, our research group has synthesized a new [Cu(NN_1_)_2_]ClO_4_ complex, where NN_1_ is a 6-((quinolin-2-ylmethylene)amine)-2H imine ligand-chrome-2-one, a derivative of the natural compound coumarin (Aldabaldetrecu et al., 2020). This complex was shown to have a better antibacterial effect *in vitro* against *F. psychrophilum* than its precursors, coumarin and copper (I) salt (Aldabaldetrecu et al., 2020), and that generates protection in *O. mykiss* when challenged with *F. psychrophilum* (Aldabaldetrecu et al., 2022). In that research, no antibacterial activity of coumarin was observed, therefore, the increase in the effect of [Cu(NN_1_)_2_]ClO_4_ compared to coumarin, was probably due to the ability of coumarin to permeate the membrane (Galkin et al., 2009), functioning as a carrier for the copper metal center of the complex. Based on these previous results, in this manuscript we show the results obtained from the evaluation of the effect of administration for 60 days of [Cu(NN_1_)_2_]ClO_4_ in *O. mykiss* on the immune system and its ability to modulate the composition of the intestinal microbiota.

## 2 Materials and methods

### 2.1 Synthesis of Cu (I) coordination complex

The synthesis of Cu(I) coordination compound [Cu(NN_1_)_2_]ClO_4_, where NN_1_ is the ligand 6-((quinolin-2-ylmethylene)amino)-2H-chromen-2-one was carried out by template condensation method (Cáceres-Vásquez et al., 2023). To 3 mmol of the precursor reagent [Cu(CH_3_CN)_4_]ClO_4_ dissolved in 50 mL of acetonitrile, double of the amounts of the reactants 2-quinoline-carboxaldehyde (6 mmol) and 6-amino-chromen-2-one (6 mmol), dissolved in acetonitrile, were added and maintained at room temperature and constant stirring for 1 h, forming a colored solution. The volume of solution was reduced in a rotary evaporator and the concentrate was precipitate with cold ethyl ether. The microcrystalline precipitate was recrystallized from an ethyl ether/acetonitrile (9:1) mixture and finally washed with ethyl ether. The chemical structure was confirmed by comparing the signals obtained from the NMR^1^H spectrum of the complex obtained, with what was previously reported (Aldabaldetrecu et al., 2020).

### 2.2 Fish and maintenance

Pre-smolt rainbow trout (*O. mykiss*) weighing between 10 and 15 g were used (Fish farming Federico Albert Taupp Rio Blanco, Los Andes, Chile). The fish were acclimated for 1 week before treatment at 12°C in freshwater aquariums with a biomass of 14 g/L, continuous aeration, and fed with commercial pellets (Golden Optima, Biomar, Chiloé, Chile) at 1% of body weight. The fish were maintained in freshwater with a pH between 6.6 and 7, the salinity was adjusted to 6 PSU with NaCl to prevent fungal infection, and total ammonia was maintained in a range below 0.02 mg/L. Around 70% of the water in all the aquariums was changed every day before feeding. Water parameters were monitored daily prior to and after changing the water. Feeding, changing the water, and measuring water parameters were all manually conducted. The fish were maintained in accordance with the ethical standards of the Institutional Ethics Committee of the Universidad de Santiago de Chile and the current relevant legislation. The authorization of the Ethics Committee of the Universidad de Santiago de Chile to perform experiments with fish in the project FONDEF VIU, approval number 354.

### 2.3 Experimental design in fish

*O. mykiss* (120 fish) were divided into five groups, each with 24 fish divided into two aquariums with 12 fish. Group A untreated fish (Ctrl), group B fish treated with 20 μg/g of [Cu(NN_1_)_2_]ClO_4_ (20), group C fish treated with 40 μg/g of [Cu(NN_1_)_2_]ClO_4_ (40), group D fish treated with 60 μg/g of [Cu(NN_1_)_2_]ClO_4_ (60), group F fish treated with 75 μg/g of the oxytetracycline (O). All fish were fed for 60 days with commercial pellet plus the respective treatments, which were mixed using commercial oil, while the control fish were only fed with the commercial pellet mixed with oil. The mixture between pellet, copper complex and oil was carried out by mechanical agitation between 45 and 60 s. Three fish were sacrificed per aquarium at 15, 30, 45, 60, days of experimentation (*n* = 6 per treatment), and head kidney and intestine of the fish were removed. In teleost, the spleen has a prime role in the antigen presentation and initiation of the adaptive immune response. Furthermore, the anterior kidney is the main immune organ accountable for antigen processing, phagocytosis, IgM, and immune memory formation (Mokhtar et al., 2023). To sacrifice the fish, they were exposed to 30 mg/L of benzocaine in deionized water for 5–8 min. The samples were stored at −80°C. Biomar supplied the antibiotics used for the experiments. The amount of antibiotic was calculated with respect to the active ingredient.

### 2.4 RNA extraction and RT-qPCR

To extract RNA, approximately 30 mg of kidney was homogenized in 1 mL of TRIzol reagent (Invitrogen) and incubated for 5 min at room temperature, then 200 μL of cold chloroform were added and vortexed for 15 s. The samples were incubated for 3 min at room temperature and centrifuged at 12.000 *g* for 15 min at 4°C. The upper phase was recovered and precipitated with 500 μL of isopropanol, subsequently incubated for 90 min at −80°C, and centrifuged at 12.000 *g* for 10 min at 4°C. The supernatant was removed, and the pellet resuspended in 1 mL of ethanol 75% in DEPC water. The samples were centrifuged at 7.500 *g* for 5 min at 4°C. Finally, the pellet was allowed to dry for 15 min and dissolved in 40 μL of DEPC water. The RT reaction was performed with the All-In-One 5X RT MasterMix kit (abm), using 2 µg of RNA, 4 µL of the master mix and nuclease-free H_2_O to a final volume of 20 µL. The thermal profile used was 30 min at 37°C, 10 min at 60°C and 3 min at 95°C. Real-time quantitative PCR reactions (qPCR) were performed in 96-well plates (Thermoscientific) using the PikoReal 96 Real-Time PCR System (Thermoscientific). The reaction mixture consisted of 5 μL of SsoAdvanced universal^™^ SYBR^®^ Green Supermix (Biorad), 0.5 μM of forward and reverse primers for each analyzed cytokine, 80 ng of cDNA, and ultrapure water (Invitrogen) to complete a final volume to 10 μL. Subsequently, the transcript levels of the genes (IL-12, IFN-γ, IL-1β, TNF-α, TGF-β, CD4, Perforin and Lysozyme) were quantified. The thermal profile used was one cycle at 95°C for 2 min, 40 cycles at 95°C for 5 s, 60°C for 15 s, and 72°C for 15 s. For the analyses, the expression of elongation factor 1α (ef1a) was used to normalize the expression of target genes using the 2^−ΔΔCT^ method (Pfaffl, 2001), the graphs were made with Log_2_ (2^−ΔΔCT^) to better visualize the increase and decrease in the transcript levels of each gene under study. Statistically significant differences with respect to the control were determined by a one-way nonparametric *t*-test (Mann–Whitney) (*p < 0.05, **p < 0.01, ***p < 0.001). The primers used in these experiments are listed in Supplementary Table S1.

### 2.5 DNA extraction and metagenomics analysis

DNA was extracted from 30 mg of the intestine using the Wizard^®^ Genomic DNA Purification Kit (Promega) following the manufacturer’s instructions. Total DNA was then quantified by UV spectrophotometry using a Tecan INFINITE M200 Pro. Each DNA was standardized to a concentration of 25 ng/μL and a pool of three fish per aquarium (2 pools per condition) was made.

### 2.6 16S ribosomal sequencing

The 16 S rRNA amplification and high-throughput sequencing was performed by Molecular Research LP (MR DNA; Shallowater, TX, United States). Briefly, the DNA extracted from intestine samples pooling DNA from the intestine of three fish per tank per treatment was used as template for amplification of the V4 variable region of the 16 S rRNA gene. The amplification was performed using the primers 515-F and 806-R (Caporaso et al., 2011) with a barcode on the forward primer and the HotStarTaq Plus Master Mix Kit (Qiagen, United States). The PCR conditions were 94°C for 3 min, followed by 30 cycles of 94°C for 30 s, 53°C for 40 s and 72°C for 1 min, followed by a final elongation step at 72°C for 5 min. After amplification, PCR products were checked in a 2% agarose gel to determine the success of amplification and the relative intensity of bands. PCR products were purified using Ampure XP beads and used to prepare an Illumina DNA library with a TruSeq Nano kit. High-throughput sequencing of 16 S rRNA amplicons was performed with the MiSeq reagent kit v3 on the Illumina MiSeq platform (2 × 300-bp paired ends [PE]) following the manufacturer’s guidelines.

### 2.7 Bioinformatic analysis

The data derived from sequencing were processed using QIIME2 version 2020.2 for 16S-based microbiota analyses (Bolyen et al., 2019) and the construction of the pipeline was carried out based on the documents present on the QIIME2 webpage (https://docs.qiime2.org/2020.2/tutorials/). Initially, barcodes and adapters were removed from the demultiplexed paired-end sequences. For quality filtering and feature (Amplicon Sequence Variants (ASV)) prediction, we used DADA2 (Callahan et al., 2016). Forward and reverse reads were each truncated to 250 nts. Representative sequences were aligned using MAFFT (Multiple Alignment using Fast Fourier Transform) (Katoh and Standley, 2013). A phylogenetic tree of the aligned sequences was elaborated with FastTree 2 (Price et al., 2010). ASVs/features were taxonomically classified using a pre-trained Naive Bayes taxonomy classifier, Greengenes 13_8 99% ASVs (DeSantis et al., 2006). Tables of taxonomic counts and percentages (relative frequency) were generated. We obtained a mean of 27,757 individual sequencing reads per sample (min = 2,959; max = 48,403). After data processing, the average number of sequences for each sample passing through ASV classification was 161294 (SD: 115855). The average number of ASVs per sample was 27758 (SD: 22034). Rarefaction was used to sample the same number of random reads from each sample for the diversity analyses. The sampling depth was set at 26200 sequences per sample. Taxon-level abundance data were filtered to remove very low-abundance taxa (<0.05%) and taxa not represented in at least half the samples before further analysis.

## 3 Results

### 3.1 Synthesis of Cu (I) coordination complex

**FIGURE 1:**
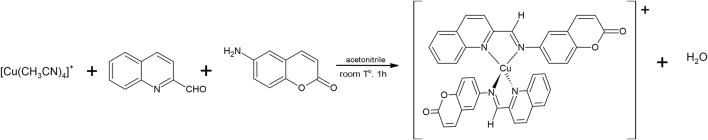
Scheme of the synthesis of the complex [Cu (NN_1_)_2_] ClO_4_ by the template method.

We have synthesized [Cu(NN_1_)_2_]ClO_4_ from the synthetic modification of the natural product coumarin to obtain N,N′-bicoordinating ligands capable of forming complexes that stabilize the metal ion Cu(I) and improve the antibacterial activity that they have separately, the natural product and copper (Aldabaldetrecu et al., 2020). This new Cu (I) coordination complex [Cu(NN_1_)_2_]ClO_4_, where NN_1_ = 6-((quinolin-2-ylmethylene)amino)-2H-chromen-2-one, a ligand derivate from coumarin 1-benzopyran-2-one, initially was obtained by several steps. The first step consisted in the nitration of coumarin followed by its reduction to 6-aminocoumarin with Fe powder. The last step consisted in the condensation reaction of this amine with two-quinoline carboxaldehyde by microwave-assisted reaction to give the imine ligand NN_1_ as product. This process takes around 12 h. Finally, the reaction of NN_1_, with [Cu(CH_3_CN)_4_]ClO_4_ precursor complex under mild reaction conditions generates the Cu (I) coordination complex as reported by Aldabaldetrecu et al. (2020). Although the reaction was obtained with a 76% yield, our studies continued in the search to improve the yield and minimize the reaction time. It is so, in this new research, the synthesis was easily by template condensation method from equimolar amounts of reagents as shown in Figure 1, which allowed that the reaction time to be reduced to1hour, with 90% yield, at room temperature and without requiring pressure systems, substantially improving the energy and economic cost of the process.

### 3.2 Effect of [Cu(NN_1_)_2_]ClO_4_ on the growth of *Oncorhynchus mykiss*

**FIGURE 2:**
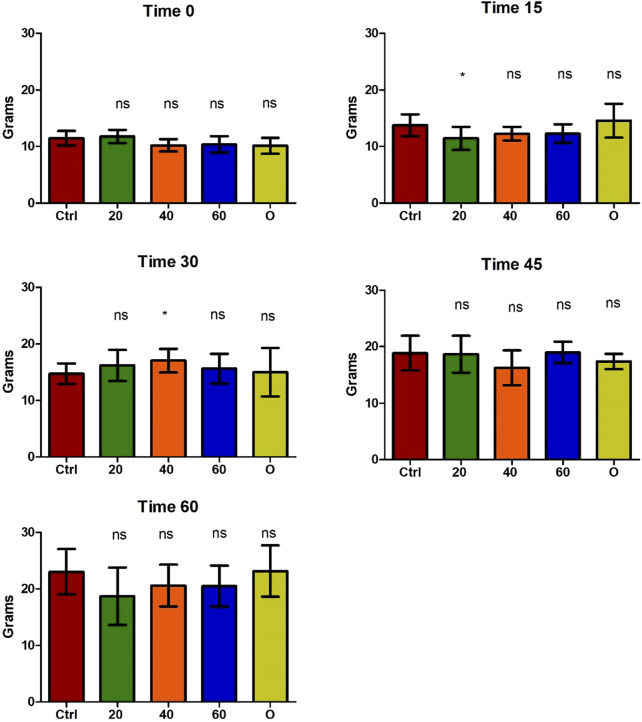
Growth of *Onchorhynchus mykiss* during the 60 days of experimentation. Every 15 days, six fish per condition were sampled. Ctrl (control), 20 ([Cu(NN_1_)_2_]ClO_4_, 20 μg/g of fish), 40 ([Cu(NN_1_)_2_]ClO_4_, 40 μg/g of fish), 60 ([Cu(NN_1_)_2_]ClO_4_, 60 μg/g of fish), O (oxytetracycline, 75 μg/g of fish). The significance was analyzed using the Mann Whitney test (*p < 0.05), ns, non-significant difference.

The safety of the administration of [Cu(NN_1_)_2_]ClO_4_ for 60 days in *O. mykiss* was evaluated. During feeding, no negative effects on salmonid behavior were observed. Similarly, no changes were observed in the growth of the salmonids during the 60 days of experimentation (Figure 2).

### 3.3 Evaluation of the administration of [Cu(NN_1_)_2_]ClO_4_ on immune status of *Oncorhynchus mykiss*

**FIGURE 3:**
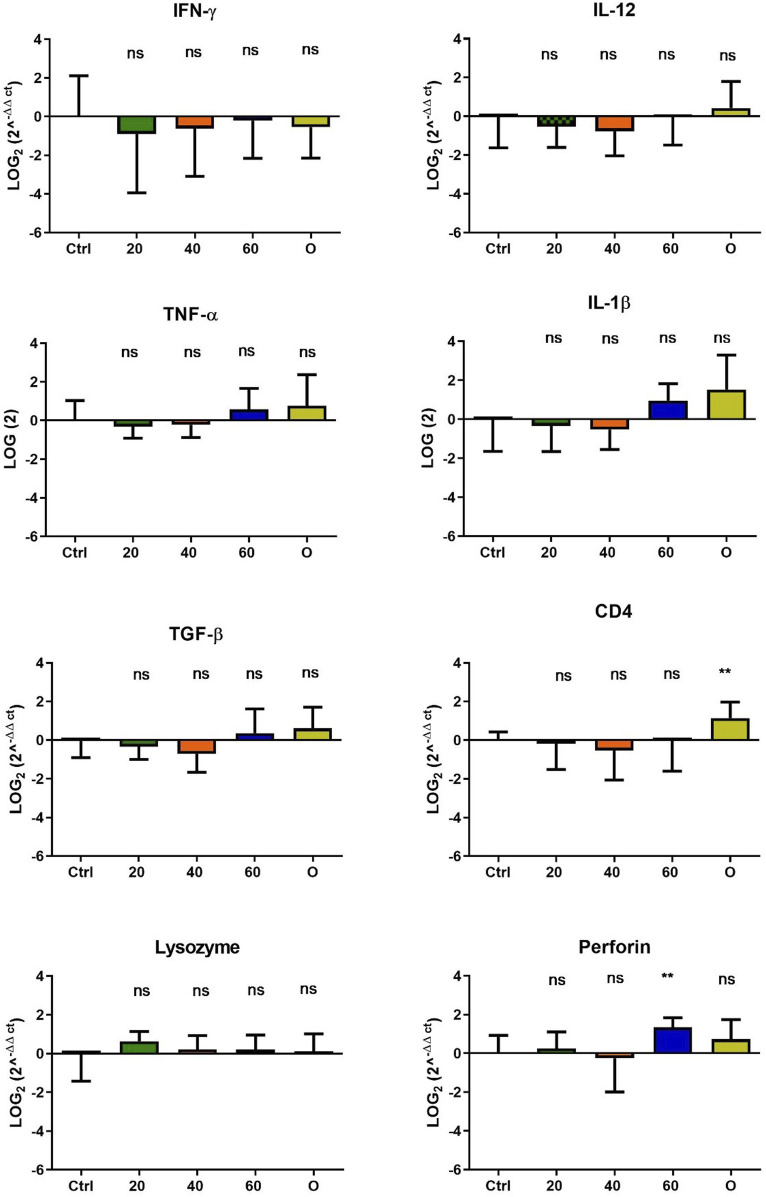
Effect of feeding *Onchorhynchus mykiss* with [Cu(NN_1_)_2_]ClO_4_ for 15 days on the immune system. The figure shows the transcript levels of 8 markers of the cellular immune response. Ctrl (control), 20 ([Cu(NN_1_)_2_]ClO_4_, 20 μg/g of fish), 40 ([Cu(NN_1_)_2_]ClO_4_, 40 μg/g of fish), 60 ([Cu(NN_1_)_2_]ClO_4_, 60 μg/g of fish), O (oxytetracycline, 75 μg/g of fish). The significance was analyzed using the Mann Whitney test (*p < 0.05), ns, non-significant difference.

**FIGURE 4:**
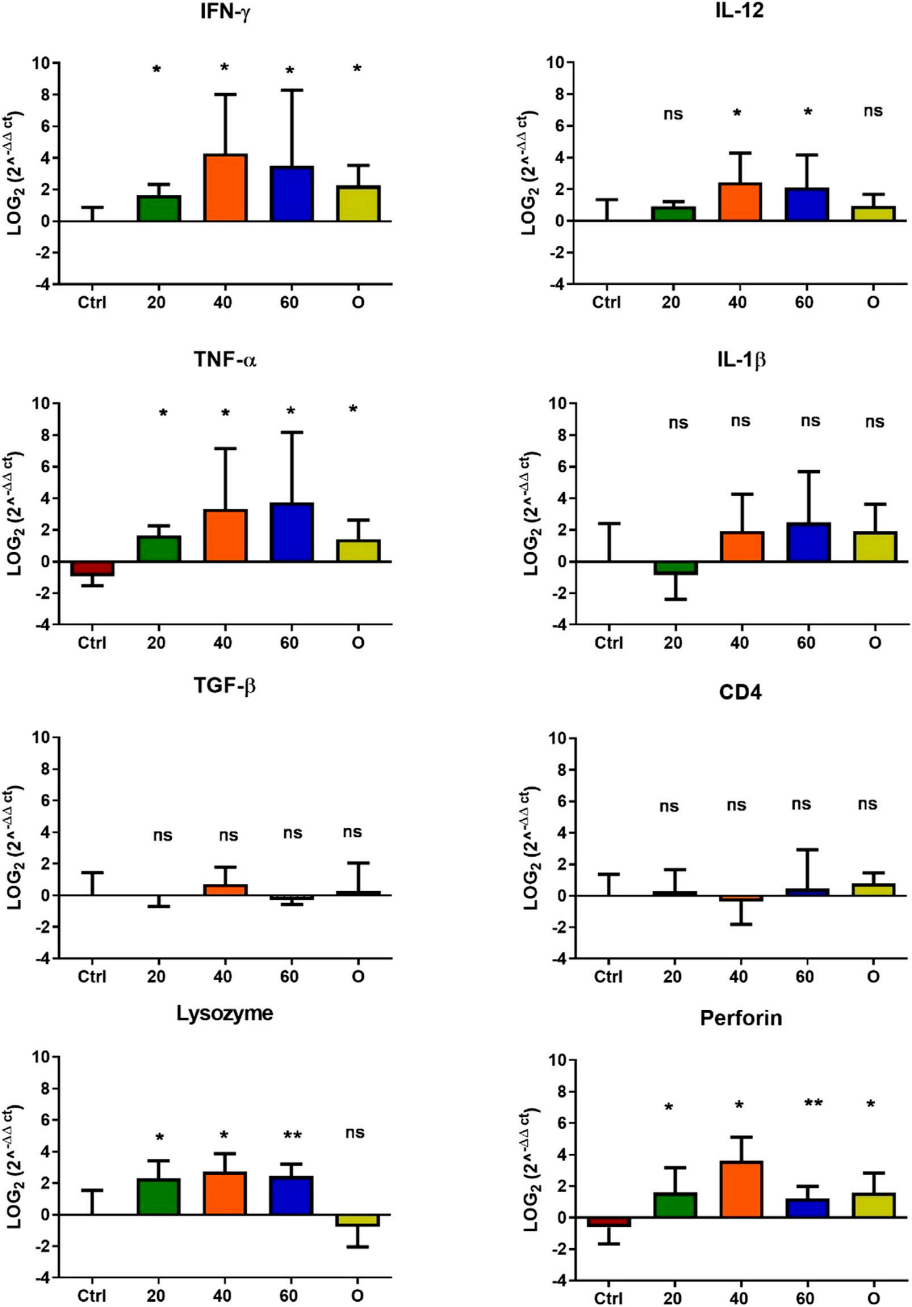
Effect of feeding *Onchorhynchus mykiss* with [Cu(NN_1_)_2_]ClO_4_ for 30 days on the immune system. The figure shows the transcript levels of 8 markers of the cellular immune response. Ctrl (control), 20 ([Cu(NN_1_)_2_]ClO_4_, 20 μg/g of fish), 40 ([Cu(N_1_)_2_]ClO_4_, 40 μg/g of fish), 60 ([Cu(NN_1_)_2_]ClO_4_, 60 μg/g of fish), O (oxytetracycline, 75 μg/g of fish). The significance was analyzed using the Mann Whitney test (*p < 0.05), ns, non-significant difference.

**FIGURE 5:**
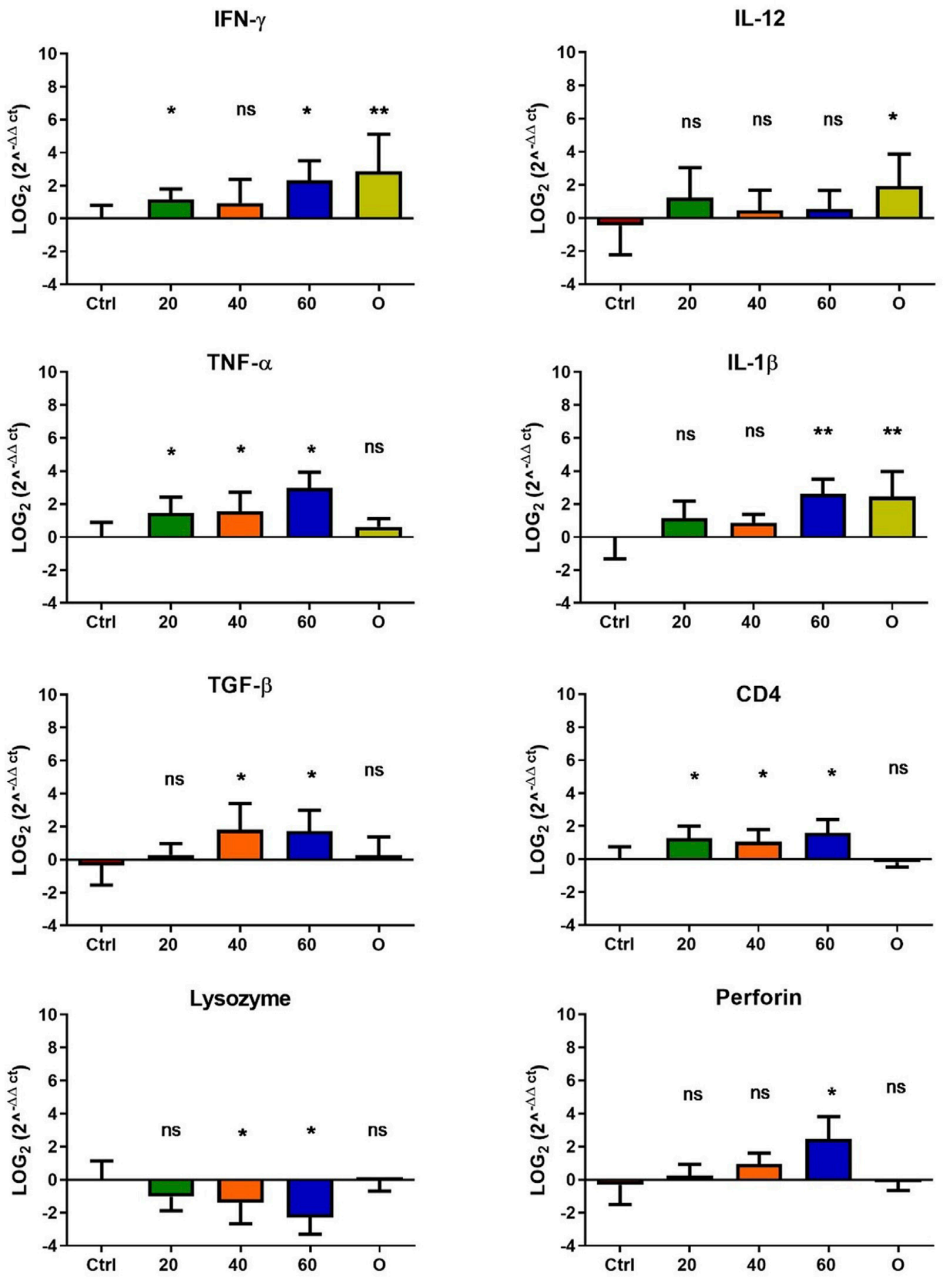
Effect of feeding *Onchorhynchus mykiss* with [Cu(NN_1_)_2_]ClO_4_ for 45 days on the immune system. The figure shows the transcript levels of 8 markers of the cellular immune response. Ctrl (control), 20 ([Cu(NN_1_)_2_]ClO_4_, 20 μg/g of fish), 40 ([Cu(NN_1_)_2_]ClO_4_, 40 μg/g of fish), 60 ([Cu(NN_1_)_2_]ClO_4_, 60 μg/g of fish), O (oxytetracycline, 75 μg/g of fish). The significance was analyzed using the Mann Whitney test (*p < 0.05), ns, non-significant difference.

**FIGURE 6:**
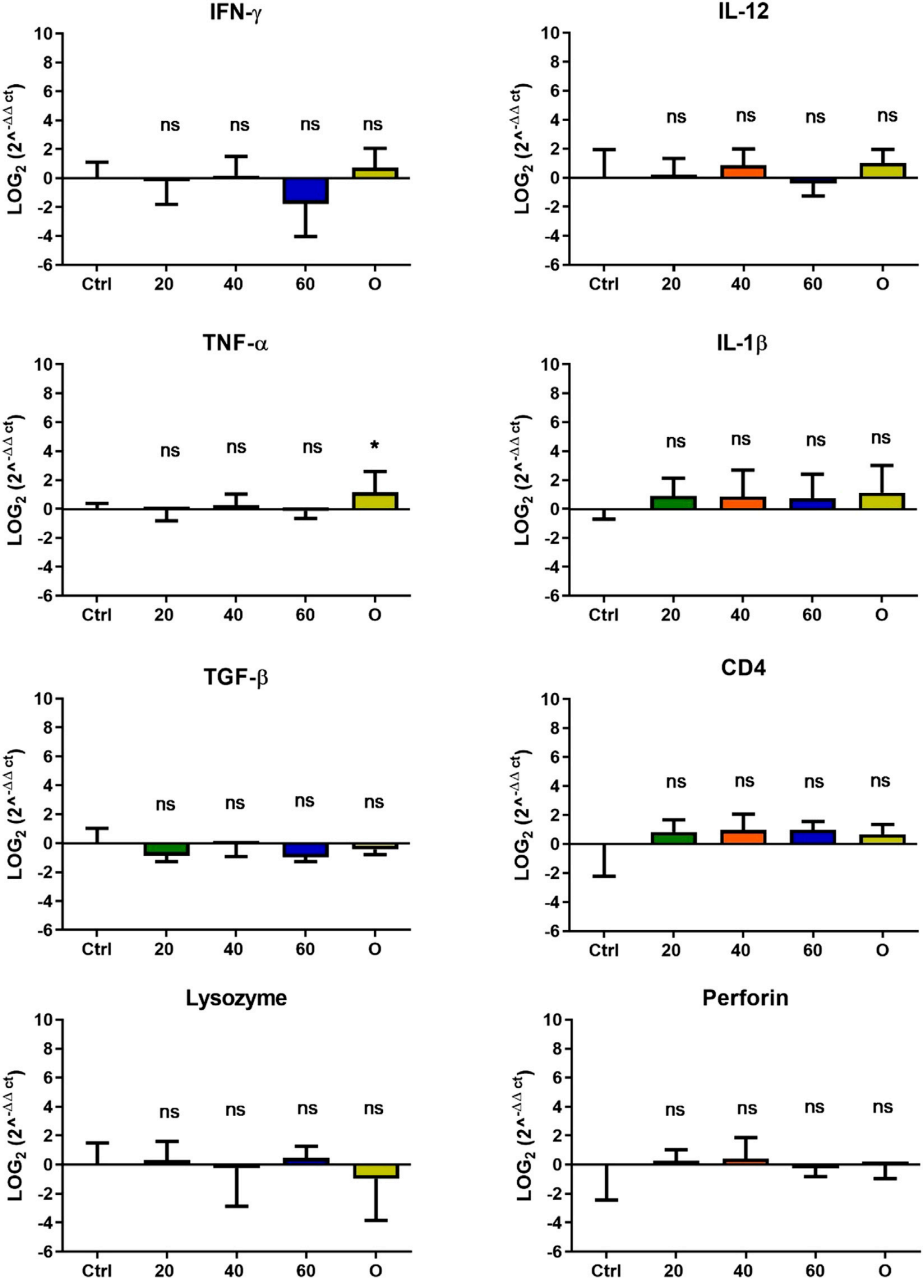
Effect of feeding *Onchorhynchus mykiss* with [Cu(NN_1_)_2_]ClO_4_ for 60 days on the immune system. The figure shows the transcript levels of 8 markers of the cellular immune response. Ctrl (control), 20 ([Cu(NN_1_)_2_]ClO_4_, 20 μg/g of fish), 40 ([Cu(NN_1_)_2_]ClO_4_, 40 μg/g of fish), 60 ([Cu(NN_1_)_2_]ClO_4_, 60 μg/g of fish), O (oxytetracycline, 75 μg/g of fish). The significance was analyzed using the Mann Whitney test (*p < 0.05), ns, non-significant difference.

In *O. mykiss*, the administration of [Cu(NN_1_)_2_]ClO_4_ generated a greater stimulation of the immune system markers analyzed. Although during the first 15 days of feeding, only the administration of 60 μg/g of fish generated an increase close to 2 times respect to the control in the levels of perforin transcript (Figure 3), at 30 and 45 days of feeding, greater changes were noticed due to feeding with the [Cu(NN1)2]ClO4. After 30 days of continuous feeding with [Cu(NN_1_)_2_]ClO_4_, an increase close to 16-fold in IFN- γ and TNF-α transcript levels was observed in fish treated with 60 μg/g of fish, while an increase close to 4-fold in IL-12 and lysozyme transcript levels. Finally, a 2-fold increase in perforin transcript levels was also observed (Figure 4). On the other hand, in fish fed 40 μg/g of fish, a 16-fold increase in the levels of IFN- γ and TNF-α transcript was also observed, while the levels of IL-12 and lysozyme transcripts increased 4-fold. Likewise, a 8- to16-fold increase in perforin transcript levels was observed (Figure 4). Furthermore, in fish fed 20 μg/g of fish, a 2- to 4-fold increase in IFN- γ, TNF-α, peroforine and lysozyme transcript levels was also observed (Figure 4). Finally, fish that were fed with oxytetracycline also observed an increase in transcript levels of immune markers, but to a lesser extent. In the case of IFN-γ transcript levels, a 4-fold increase was observed, while TNF-α and perforin transcript levels increased 2-fold (Figure 4). After 45 days of continuous feeding with [Cu(NN_1_)_2_]ClO_4_, in fish treated with 60 μg/g of fish, a two to 4-fold increase in the transcript levels of IFN-γ, TNF-α, IL-1β, TGF-β and perforin was observed, while CD4 transcript levels increased 2-fold, and lysozyme transcript levels decreased 4-fold (Figure 5). Furthermore, fish fed 40 μg/g of [Cu (NN_1_)_2_]ClO_4_ showed a 2- to 4-fold increase in TNF-α, TGF-β and CD4 transcript levels. In contrast, lysozyme transcript levels decreased by 2-fold (Figure 5). On the other hand, fish fed 20 μg/g of [Cu (NN_1_)_2_]ClO_4_ showed a 2-fold increase in IFN-γ, TNF-α, and CD4 transcript levels (Figure 5). Furthermore, fish treated with oxytetracycline also showed a 4-fold increase in IFN-γ, IL-12 and IL-1 β transcript levels (Figure 5). Finally, at 60 days of experimentation, only fish treated with oxytetracycline showed a 2-fold increase in TNF-α transcript levels (Figure 6).

### 3.4 Effect of the administration of [Cu(NN_1_)_2_]ClO_4_ on the composition of the intestinal microbiota of *Oncorhynchus mykiss*

**FIGURE 7:**
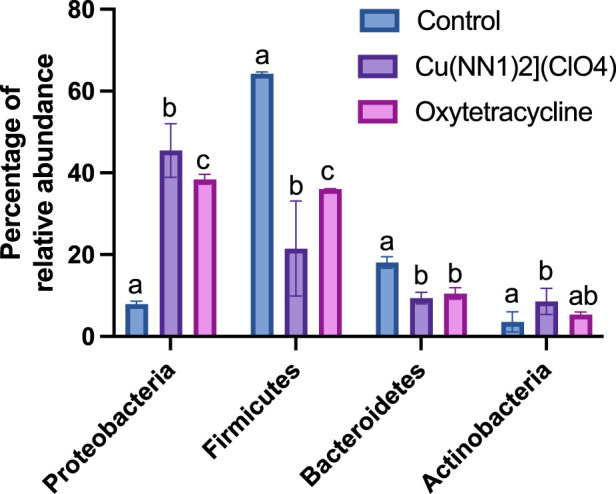
Effect of [Cu(NN_1_)_2_]ClO_4_ complex and oxytetracycline on the composition of the intestinal microbiota of *Onchorhynchus mykiss*. The figure shows the relative abundance of the Phylum that show statistically significant differences (*p* < 0.05) in their relative abundance in control fish (*n* = 2), treated with [Cu(NN_1_)_2_]ClO_4_ (*n* = 4) and treated with oxytetracycline (*n* = 2). Statistical analysis was performed with a 2-way ANOVA. Different letters mean statistically significant differences.

**FIGURE 8:**
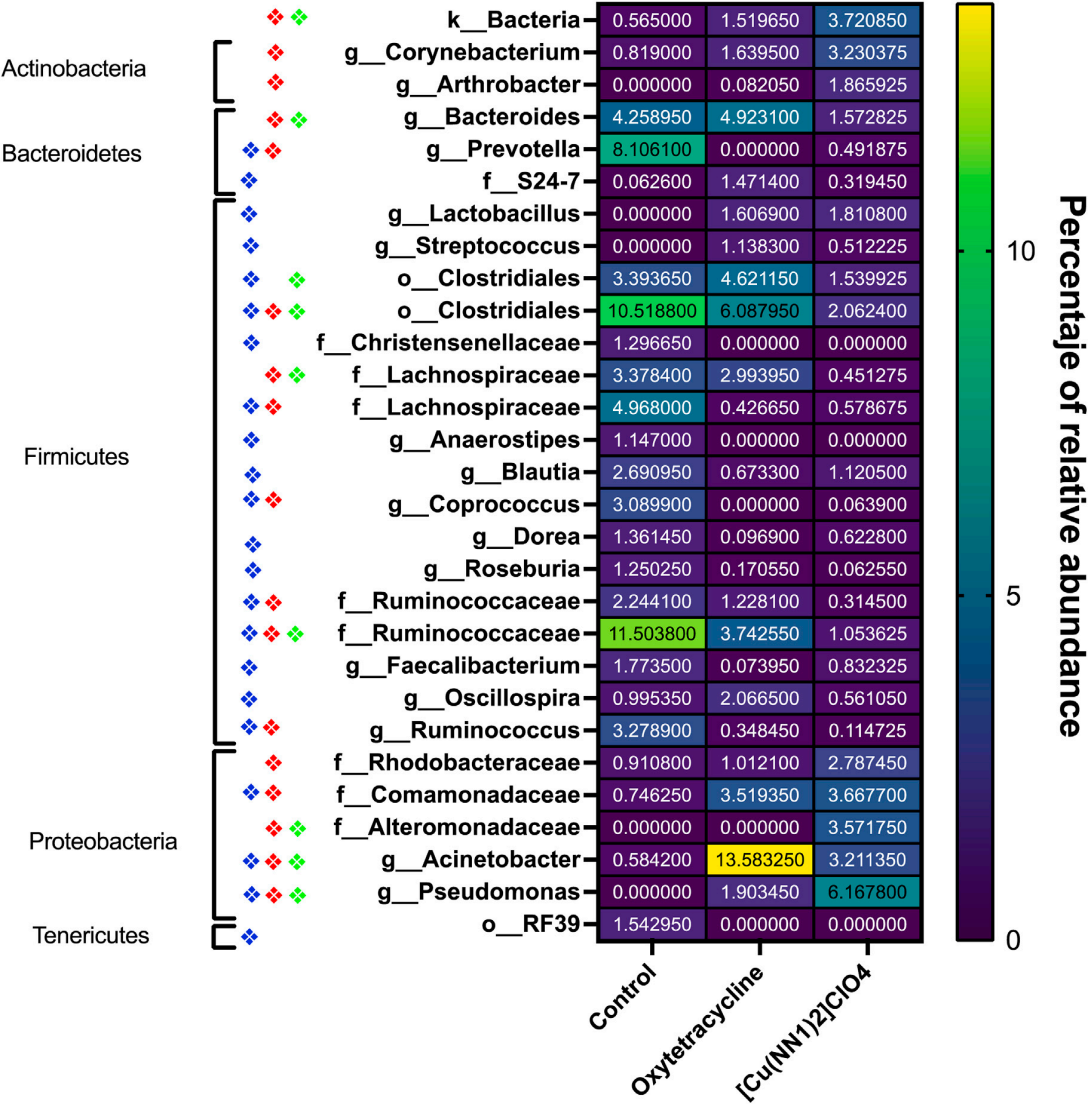
Heat-Map of taxa of the intestinal microbiota of *Onchorhynchus mykiss* affected by the administration of [Cu(NN_1_)_2_]ClO_4_ and oxytetracycline. The figure shows the taxa present in the intestinal microbiota of *Onchorhynchus mykiss* specimens fed with control food (*n* = 2), oxytetracycline (*N* = 2) and [Cu(NN_1_)_2_]ClO_4_ (*N* = 2) whose relative frequency underwent changes statistically significant (*p* < 0.05). The diamonds represent the presence of a statistically significant difference (*p* < 0.05) between Taxa of the control fish and the fish treated with oxytetracycline (Blue Diamond), between the control fish and the fish treated with [Cu(NN_1_)_2_]ClO_4_ (Red Diamond), and between fish treated with oxytetracycline and fish treated with [Cu(NN_1_)_2_]ClO_4_ (green diamond). The average relative frequency of each taxa in each condition is shown on a color scale, and indicated numerically in the center of each box. The figure also indicates the Phylum to which each of the identified taxa belongs. The graph and statistical analyzes were performed with the GraphPad 8.0 program.

**FIGURE 9:**
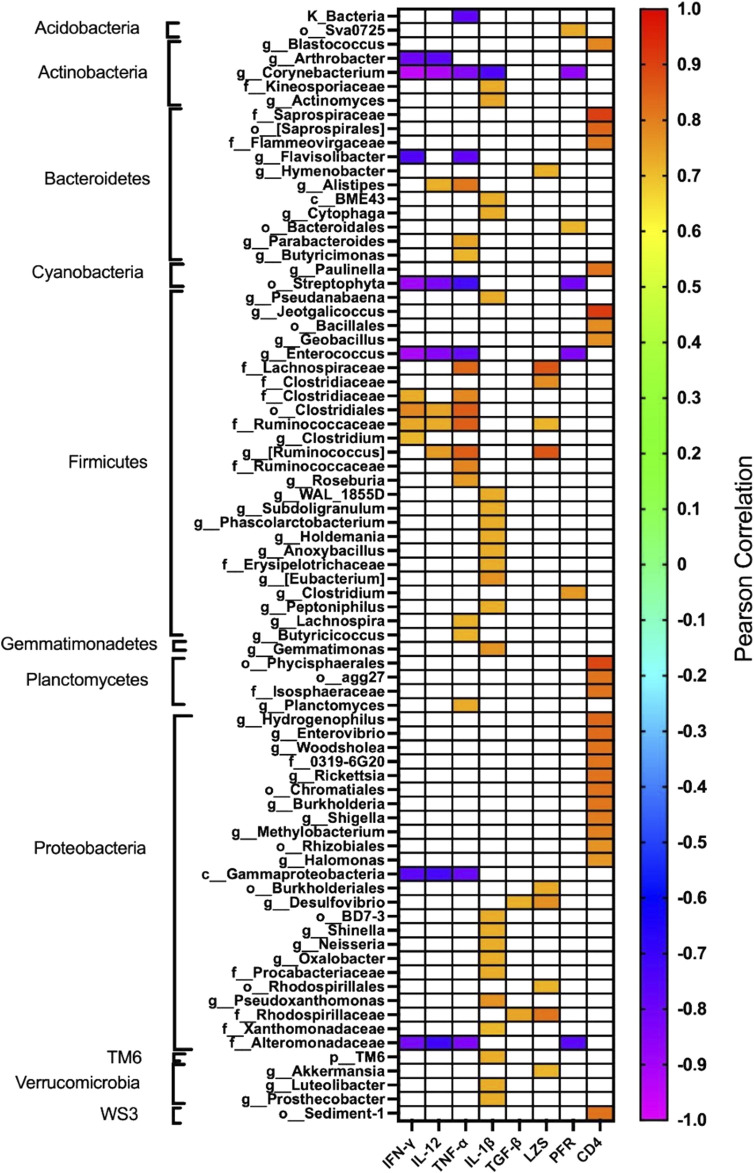
Correlation between the relative abundance of each taxa and the immune status of the fish. The figure shows the correlations between the relative abundance of each of the taxa identified in fish treated with normal food (*n* = 2), [Cu(NN_1_)_2_]ClO_4_ 40 μg/g (*n* = 2), [Cu(NN_1_)_2_]ClO_4_ 60 μg/g, (*n* = 2) and oxytetracycline (*n* = 2) with the expression levels of interferon gamma (IFN-γ), Interleukin 12 (IL-12), TNF-alpha (TNF-α), Interleukin 1beta (IL-1β), TGF-beta (TGF-β), Lysozyme (LZS), perforin (PFR), and the CD4 receptor. The expression levels were entered with ΔCt values normalized with respect to the expression of the housekeeping gene, thus positive correlations represent a decrease in expression when the relative abundance of the taxa increases. The sample color scale represents the Pearson correlation coefficient values, with *p* < 0.05. Correlation coefficients with *p* > 0.05 are indicated as white boxes. The taxa were ordered according to their classification at the Phylum level. The graph was made using the GraphPad 8.0 program.

The effect of oral administration of the Cu (I) complex on the intestinal microbiota was evaluated from intestine samples of *O. mykiss* specimens fed for 15 days with 40 and 60 μg/g of fish. The 30-day samples were chosen because it was the time where a greater effect was observed on the markers of the immune system analyzed. The same reason was used to choose the concentrations analyzed. As a control, fish fed with a normal diet or supplemented with oxytetracycline were used. In *O. mykiss* fed with the control diet, an average of 131.5 ± 5.5 ASVs were identified, distributed mainly in the phylum Firmicutes (64.2% ± 0.2%), Bacteroidetes (18.1% ± 1.0%), Proteobacteria (7.9% ± 0.5%) and Actinobacteria (3.5% ± 1.8%) (Figure 7, Supplementary Material S1). The ASV with the highest abundance (11.5% ± 0.6%) belongs to the Ruminococcaceae family (Supplementary Material S1). The administration of the compound produced an important modification of the composition of the intestinal microbiota, increasing the prevalence of bacteria belonging to the phylum Proteobacteria (45.5% ± 6.5%) and Actinobacteria (8.5% ± 3.2%), and decreasing the relative proportion of Firmicutes (21.5% ± 11.6%) and Bacteroidetes (9.4% ± 1.4%) (Figure 7). At the genus level, the compound increased the relative proportion of ASV from members of the Comamonadaceae family (3.7% ± 1.4%) and of Gammaproteobacteria of the Alteromonadaceae family (3.6% ± 4%), and of the *Pseudomonas* genus (3.1% ± 2.97%) and from one ASV that cannot be assigned to any known phylum (3.7% ± 1.96%) (Figure 8; Supplementary Material S1). When the fold changes are compared, the two ASVs that increase their abundance the most, approximately 32-fold, are the ASVs classified as members of the class Alphaproteobacteria and the ASVs assigned to the genus *Corynebacterium*. On the other hand, the bacteria that showed the greatest reduction (approximately 64 times) in their abundance, they belong to the genera *Coprococcus* and *Ruminococcus* (Supplementary Material S1). When these changes are compared with those produced by oxytetraclyne, it was observed that the Cu (I) complex more effectively increases the relative proportion of Proteobacteria and Actinobacteria and shows a more pronounced effect on the decrease in the relative abundance of bacteria belonging to the phylum Firmicutes. At the genus level, the microbiota of fish treated with the antibiotic show as dominant ASVs bacteria belonging to the genus *Acinetobacter* (13.1% ± 3.5%) and the order Clostridiales (6.1% ± 0.8%), with ASVs classified as belonging to the phylum firmicutes being those that increased their abundance around 32-fold. On the other hand, ASV classified as belonging to the genera *Ruminococcus* and *Roseburia* showed a decrease of around 16-fold in their relative abundance (Supplementary Material S1). When the effect of [Cu(NN_1_)_2_]ClO_4_ was compared at the genus level, it was observed that 17 genera presented statistically significant differences in their relative abundance when the microbiota of control fish was compared with the microbiota of fish treated with [Cu(NN_1_)_2_]ClO_4_. Most of these genera belong to the phylum Firmicutes, however, ASV classified within the genus *Pseudomonas* were those that experienced a greater increase in their relative abundance (Figure 8). To evaluate whether the changes observed in the composition of the microbiota are related to the gene expression levels of IFN-γ, TNF-α, IL-1β TGF-β, IL-12, lysozyme, perforin and CD4, correlations were sought between the Ct values of each of these normalized genes by the Ct value of the houskeeping gene in the head kidney of control fish, fish treated with antibiotics and fish treated with [Cu(NN_1_)_2_]ClO_4_ at 40 and 60 μg/g of fish. Using this approach, strong correlations (*p* < 0.05, r > 0.7) were found between the levels of IFN-γ, IL-12, and TNF-α, and the relative abundance of some microorganisms belonging to the Clostridiaceae and Ruminococcaceae families. These genes also showed negative correlations (*p* < 0.05, r > 0.7) with the abundance of microorganisms belonging to the genera *Corynebacterium, Arthrobacter, Flavisolibacter, Enterococcus*, and a member of the Alteromonadaceae family and a member belonging to the Gammaproteobacteria class. In the case of IL-1β, this gene only showed positive correlations (*p* < 0.05, r > 0.7), with several members belonging to the Phylum, Actinobacteria, Bacteroidetes, Firmicutes, Gemmatimonadetes, Proteobacteria, TM6 and Verrumicrobia, being the bacteria belonging to the Phylum Firmicutes the most abundant. In the case of TGF-β, the expression of this gene was only positively correlated with bacteria of the genus *Desulfovibrio* and the family Rhodospirillaceae. Lysozyme gene expression was also correlated with members of the Lachnospiraceae and Clostridiaceae families, while perforin expression was also correlated with some members of the Proteobacteria and Firmicutes families. The most different correlation pattern was found in the expression of CD4, which showed positive correlations with members of the Phylum Bacteroidetes, Cyanobacteria, Firmicutes, Planctomycetes, Proteobacteria and WS3, which did not show correlation with any of the ASVs associated with the expression of the other genes analyzed. Considering that a positive correlation implies that a higher relative proportion of ASV is associated with a higher delta Ct value and that this, in turn, implies lower gene expression, it is inferred that most of the microorganisms identified reduce gene expression analyzed, with the exception of the effect of *Corynebacterium*, *Arthrobacter* or *Enterococcus* which would stimulate the expression of the genes associated with the expression of IFN-α, IL-12, TNF-α and perforin (Figure 9).

## 4 Discussion

The aquaculture industry in Chile is one of the most important economic sectors. However, the rapid growth of this productive sector has generated the continuous appearance of pathogens that affect the different production species. To face this problem, the Chilean aquaculture industry has used a large amount of antibiotics, generating various environmental problems and devaluing the Chilean product. In search of new alternatives, our working group synthesized the Cu (I) complex [Cu(NN_1_)_2_]ClO_4_) using coumarin as ligand, managing to improve the antibacterial capacity against *F. psycrhophilum* than its precursor molecules (coumarin and [Cu(CH_3_CN)_4_]ClO_4_) (Aldabaldetrecu et al., 2020) and generating protection for *O. mykiss* against a challenge with *F. psycrhophilum* (Aldabaldetrecu et al., 2022). Taking this background into account, and although the compound was initially designed and evaluated as an antibacterial, in this work we study the ability of [Cu(NN_1_)_2_]ClO_4_ to stimulate the cellular immune response in *O. mykiss*, and its relationship with the modulation in the composition of the intestinal microbiota. In this way, it complements the possible mechanism of action by which this compound can generate protection in salmonids against a challenge with pathogenic bacteria. In addition, in this work we describe synthetic modifications that allowed the complex to be obtained in less time and with a higher yield, due to the use of the template method. The advantage of the template condensation method is that it involves two main effects that favor the reaction. First, the kinetic template effect, in which the metal ion Cu (I) is able to hold two reactive functions in a position that allows a reaction to occur favours an intramolecular cyclization. Then, the thermodynamic template effect, where the complexation to Cu (I), benefits the production of a thermodynamically more stable product from the reaction (Costes et al., 2013). This allowed that the reaction time to be reduced to 1 h, at room temperature and without requiring pressure systems.

In fish, the main immune organ is the kidney, which is responsible for the differentiation and activation of the different cell types. We have previously reported that the imbalance in the composition of the intestinal microbiota is capable of modifying the immune response in the kidney (Parra et al., 2020). Demonstrating that there is a close relationship between the different organs of fish, and that a local effect can have repercussions at a systemic level. Considering this background, in this work we evaluate the effect of the administration of [Cu(NN1)_2_]ClO_4_ and oxytetracycline through food, on the composition of the intestinal microbiota and its impact on the systemic immune response, evaluating the transcript levels of the genes of interest in anterior kidney.

The administration of [Cu(NN_1_)_2_]ClO_4_ in *O. mykiss* increased the transcript levels of all immune system markers analyzed. The transcript levels of the genes of two proteins that play important functions in the cellular immune response in mammals and that have also been reported in fish, such as perforin and lysozyme, were analyzed. Perforin is a glycoprotein, which in mammals is expressed by NK cells, CD8^+^ T lymphocytes and CD4^+^ T lymphocytes (Voskoboinik et al., 2010), which form pores in the cell membrane of the target cells (Lichtenheld et al., 1988). In fish, its role in bacterial and viral infections has been studied (Xu et al., 2021). For example, in rock bream, an increase in perforin is observed in the inhibition of Rock bream iridovirus (RBIV) replication (Jung and Jung, 2017). Similar results were observed in zebrafish against The Spring viraemia of carp virus (SVCV) (Varela et al., 2016), in Atlantic salmon against Pilchard orthomyxovirus (POMV) (Samsing et al., 2020) and rohu and common carp against *A. hydrophila* (Robinson et al., 2014; Li et al., 2018). On the other hand, it has been observed that stimulation with natural compounds such as nobitelin, a flavonoid, increased the production of perforin (Saito et al., 2015). Lysozyme is an enzyme with antibacterial activity that hydrolyzes the peptidoglycan layer of the bacterial cell wall by cleaving the beta-1,4 glycosidic bonds between N-acetylmuramic acid and N-acetylglucosamine (Saurabh and Sahoo, 2008; Song et al., 2021). However, lysozyme is the first line of defense against different types of pathogens such as fungi, parasites, bacteria and viruses (Buonocore et al., 2014; Wei et al., 2014; Song et al., 2021). Various studies show that feed supplementation with different secondary metabolites such as tea polyphenols increases lysozyme activity in common carp (Jahazi et al., 2020) and Asian sea bass (Ahmadi et al., 2022), as well as flavonoids in northern snakehead fish (Li et al., 2019). In this work, the transcription levels of IFN-γ, a key cytokine in the immune response of both mammals and teleost fish, were also evaluated. Various cell types such as Natural Killer (NK), CD4 + T helper (Th) cells, cytotoxic CD8^+^ cells among others, produce this cytokine (Schroder et al., 2004; Pereiro et al., 2019). The functions of IFN-γ are several, among which the activation of macrophages and CD4^+^ T helper lymphocytes has been studied, a fundamental process for eliminating intracellular pathogens, in addition to promoting inflammation and the presentation of antigens, a process for the inhibition of replication viral (Boehm et al., 1997; Green et al., 2017). On the other hand, IFN-γ activates macrophages towards the “M1″ phenotype, which increases the expression of pro-inflammatory cytokines such as IL-12, IL-1β and TNF-α, in addition to reactive oxygen species (Sica and Mantovani, 2012). In addition, IL-12, IL-1β and IL-8 promote the synthesis of IFN-γ, while TGF-β represses the expression of IFN-γ (Pereiro et al., 2019). The transcript levels of IL-12, IL-1β and TNF-α were also increased in fish treated with [Cu(NN_1_)_2_]ClO_4_, demonstrating a relationship in the ability to stimulate the cellular immune response of this compound. The relevance of IFN-γ in the immune response was also reported when measuring the effect of recombinant IFN-γ, for example, in goldfish rIFN-γ increases the transcript levels of TNF-α, IL-1β, IL-12, IL-8, CCL-1, INOS and IFN-γ, while no changes were observed in TGF-β. On the other hand, an increase in the phagocytic capacity of macrophages was also observed (Grayfer and Belosevic, 2009; Grayfer et al., 2010). Similar results were reported in *O. mykiss*, where the administration of rIFN-γ increases the transcript levels of IFN-γ, IL-6, STAT1, gamma IP10, in addition to increasing lysozyme activity. On the other hand, the administration of rIFN-γ increased the survival of salmonids against an infection with *F. psychrophilum* (Santibañez et al., 2021). Different studies show that the administration of different compounds in fish generates an increase in the expression of transcript levels of pro-inflammatory and anti-inflammatory cytokines. For example, caffeic acid and ferulic acid increase the transcript levels of IFN-γ, IL-1β and TNF-α in Nile Tilapia, generating protection against *Aeromonas veronii* (Yilmaz, 2019; Dawood et al., 2020). Trans-cinnamic acid increases IFN-γ, IL-1β, IL-8, TGF-β and TNF-α, increasing the survival of *O. mykiss* against *Yersinia ruckeri* (Yılmaz and Ergün, 2018). Coumarin increases IFN-γ levels, generating protection in zebrafish infected with spring viraemia of carp virus (SVCV) (Shen et al., 2018; Liu et al., 2019). On the other hand, copper has been used in the aquaculture industry for quite some time and is a basic component in the fish diet, due to its positive effects on the body and its antibacterial properties. However, the amount to be used is limited and depends on each species, since an excess of this metal generates negative effects on the fish (Sabry et al., 2021; Tavares-Dias, 2021). Various reports show that the positive effects of copper on the immune system depend on the concentration and that they decrease as the concentration increases (Linag et al., 2020). In beluga sturgeon, administration of copper at concentrations of 13 mg/kg of fish increases lysozyme activity but decreases at 195 mg/kg of fish (Mohseni et al., 2014). However, in our results, we did not observe that the increase in the concentration tested had a negative effect on the transcript levels of the genes analyzed, this was probably due to the fact that copper is not found as a salt, but rather as part of the complex together with the coumarin molecules. Similar results were reported with copper nanoparticles, which increase the activity of lysozyme in snow trout and *O. mykiss* with respect to copper sulfate (Afshari et al., 2021; Delavari et al., 2022). These works show that the effect of copper on fish depends on the way in which it is administered, improving its effect and decreasing the amount administered. Although in this work the effect of [Cu(NN_1_)_2_]ClO_4_ is not compared with copper sulfate, we previously reported that the antibacterial effect against *F. psychrophilum* of [Cu(NN_1_)_2_]ClO_4_ improves with respect to its precursors, administering a lower concentration of copper (Aldabaldetrecu et al., 2020). [Cu(NN_1_)_2_]ClO_4_ also demonstrated the ability to modify the intestinal microbiota composition of *O. mykiss*. In the experiment, [Cu(NN_1_)_2_]ClO_4_ in *O. mykiss* showed a broad effect on microbial diversity similar to tetracycline, with a specific impact on Firmicutes. Therefore, [Cu(NN_1_)_2_]ClO_4_ administration could potentially help shift the microbiota composition towards that observed in salmonids (Kim et al., 2021). An increase in *Pseudomonas* was identified in *O. mykiss*, which has been recognized as potential probiotics in fish (Vidal et al., 2023), with higher abundance in specimens surviving infectious processes (Valdés et al., 2020; Coca et al., 2023). On the other hand, the reduction in the production of butyric acid-producing bacteria (Clostridiales), acting as an immunomodulator, could partly explain the effect on stimulating the immune response towards an inflammatory state (Vargas et al., 2023). The assessment of a correlation between microbial composition and the expression values of immunological genes yielded interesting results, revealing microbial genera such as *Enterococcus* and *Corynebacterium*. Certain species within these genera, such as *Enterococcus faecium* (Ghazisaeedi et al., 2022), and *Corynebacterium amycolatum* (Gladysheva et al., 2022), have known immunostimulatory and probiotic properties. Future experiments are necessary to discriminate which of the immunostimulant effects are due to the direct impact of [Cu(NN_1_)_2_]ClO_4_ on the cells of the immune system, if it depends on the microbiota as in the case of Filifolinone (Parra et al., 2020), or if its effect occurs as a consequence of changes in the composition of the microbiota, favoring the abundance of microorganisms that induce adequate immunostimulation of the fish. However, given the antibacterial effect of [Cu(NN_1_)_2_]ClO_4_ and the correlations with the identified microorganisms, the effect of [Cu(NN_1_)_2_]ClO_4_ would be mediated by both microbiota-dependent and -independent mechanisms.

## 5 Conclusion

The research carried out in this work shows the first results of the immunostimulant effect of copper (I) complex [Cu(NN_1_)_2_]ClO_4_ in *O. mykiss*. According to the results obtained, our compound complex is probably capable of stimulating the cellular immune response mediated by different cell types such as NK cells or macrophage-type cells, due to the increase in the transcription levels of key cytokines such as IFN-γ, IL-12 and TNF-α. In addition, a stimulation of the Th-1 type cellular immune response is also probable, due to the increase in CD4 transcription levels. In addition, [Cu(NN_1_)_2_]ClO_4_ is capable of increasing transcript levels of lysozyme, a key enzyme in the innate defense immune response against bacterial pathogens. On the other hand, [Cu(NN_1_)_2_]ClO_4_ is also capable of modulating the composition of the intestinal microbiota of *Oncorhynchus mykiss*. Observing an increase in bacterial genera with probiotic potential such as *Pseudomonas*, and interestingly a decrease in butyrate-producing bacteria, which are anti-inflammatory. It was also possible to observe a correlation between certain bacterial genera and the increase in the expression of markers of the immune response. This may be essential to study which bacterial genera are important to stimulate the immune system. Finally, these data may shed light on the mechanism by which [Cu(NN_1_)_2_]ClO_4_ is capable of generating protection against challenges with bacterial pathogens, which was previously reported.

## Data Availability

The original contributions presented in the study are publicly available. This data can be found here: http://www.ncbi.nlm.nih.gov/bioproject/1105632.
