# Supplementary material for: Oral administration of a new copper (I) complex with coumarin as ligand: modulation of the immune response and the composition of the intestinal microbiota in Onchorhynchus mykiss
Source: Front Chem. 2024 May 14;12:1338614. doi: 10.3389/fchem.2024.1338614 (PMC11131136; doi:10.3389/fchem.2024.1338614)
Supplement: Supplementary file 2 [file Image2.pdf]

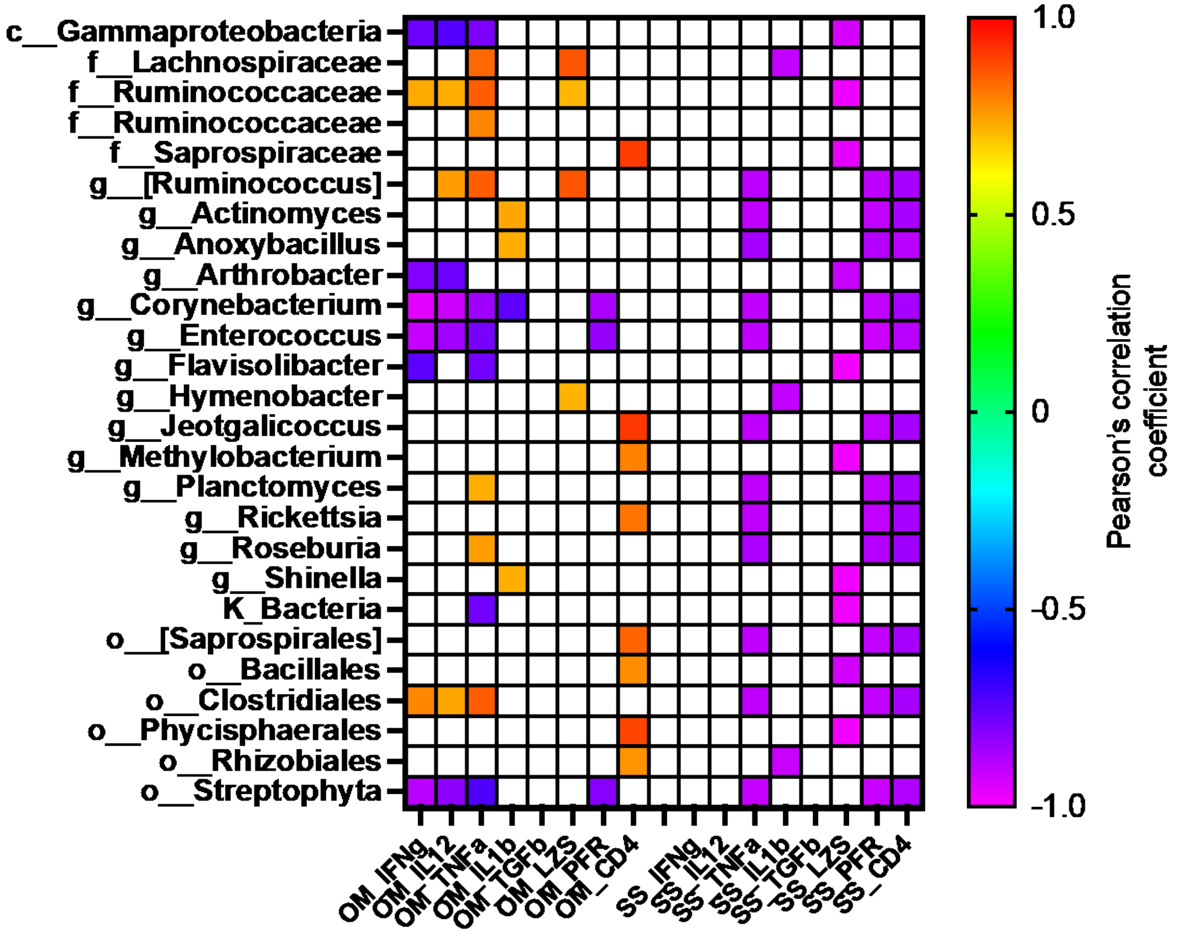

Supplementary Figure 2. ASVs of Rainbow Trout and Atlantic Salmon Correlated with Immunological Gene Expression. The figure shows the Pearson correlation index values of the ASVs identified in Atlantic Salmon and Rainbow Trout simultaneously
