# Supplementary material for: Oral administration of a new copper (I) complex with coumarin as ligand: modulation of the immune response and the composition of the intestinal microbiota in Onchorhynchus mykiss
Source: Front Chem. 2024 May 14;12:1338614. doi: 10.3389/fchem.2024.1338614 (PMC11131136; doi:10.3389/fchem.2024.1338614)
Supplement: Supplementary file 3 [file Image1.pdf]

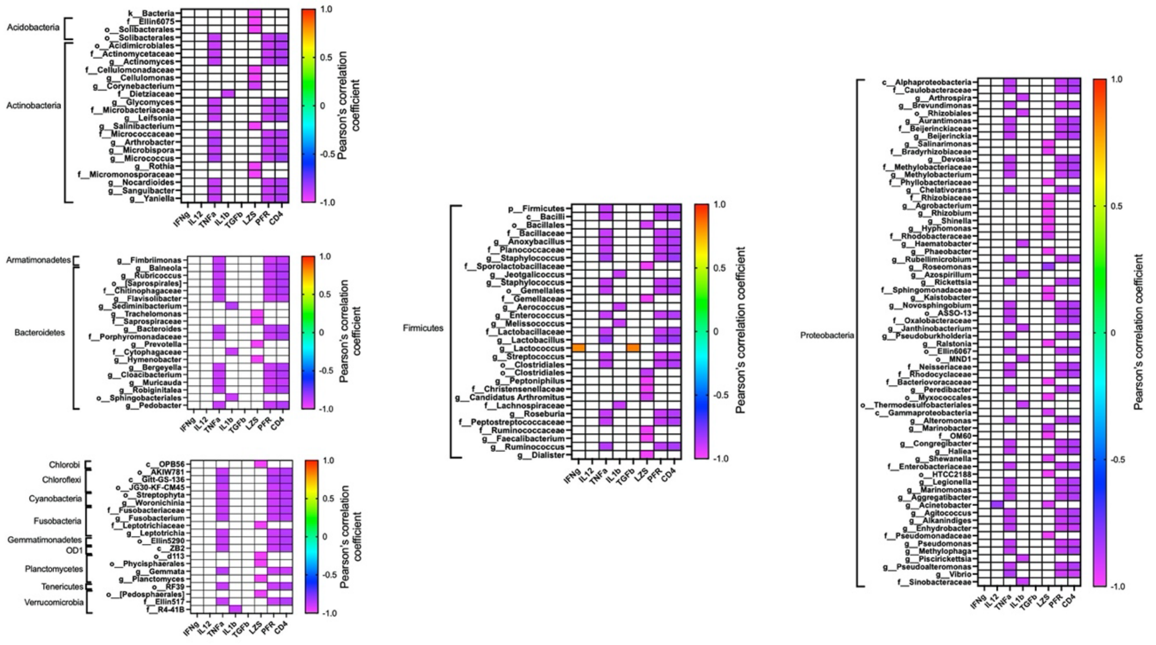

Supplementary Figure 1: Correlation between composition of the intestinal microbiota of Atlantic Salmon and the expression of immunological genes. The figure shows the Pearson correlation coefficients between the expression of immunological genes and the relative abundance of ASVs identified in the intestinal microbiota of Atlantic Salmon. The figure shows the coefficients with a p<0.05. The figure indicates the taxonomic assignment of each ASV as well as the Phylum to which it belongs.
